# Pathways from prenatal anxiety to postpartum bonding difficulties in Pakistan: A prospective longitudinal study of psychosocial mediators and the buffering role of social support

**DOI:** 10.1017/gmh.2026.10228

**Published:** 2026-05-18

**Authors:** Rakhshanda Liaqat, Kehkashan Arouj

**Affiliations:** 1 Department of Psychology, International Islamic University Islamabad, Pakistan; 2 Human Development Research Foundation Pakistan HDRF

**Keywords:** prenatal anxiety, mother–infant bonding, psychosocial stressors, social support, Pakistan

## Abstract

**Background:**

Prenatal anxiety is common and may negatively affect the early mother–infant relationship, yet it has received less attention than perinatal depression, particularly in low-resource settings. The psychosocial pathways linking prenatal anxiety to postpartum bonding difficulties remain poorly understood.

**Methods:**

This prospective longitudinal study followed 501 pregnant women in Pakistan from early pregnancy (T1; 15–20 weeks gestation) to 8 weeks postpartum (T2). Prenatal anxiety was assessed using the Generalized Anxiety Disorder Scale–7 (GAD-7), and depressive symptoms were measured with the Patient Health Questionnaire–9 (PHQ-9). Late-pregnancy psychosocial stressors, including pregnancy experiences, domestic violence, and perceived stress, as well as perceived social support, were assessed at T1.5 (30–36 weeks gestation). Postpartum outcomes included mother–infant bonding difficulties measured by the Postpartum Bonding Questionnaire (PBQ) and postpartum anxiety symptoms. Longitudinal path analysis, parallel mediation, and moderated mediation models were conducted.

**Results:**

Prenatal anxiety significantly predicted postpartum bonding difficulties (β = .18, p = .001), independent of prenatal depressive symptoms. This relationship was partially mediated by late-pregnancy psychosocial stressors (total indirect effect = 0.34, 95% CI [0.23, 0.46]). Prenatal anxiety also demonstrated continuity with postpartum anxiety (β = .45, p < .001), which was associated with poorer bonding outcomes (β = .28, p < .001). Perceived social support moderated both direct and indirect pathways. Higher support weakened the direct association between prenatal anxiety and bonding difficulties (interaction β = −.13, p = .001) and reduced indirect effects through psychosocial stressors (index of moderated mediation = −0.07, 95% CI [−0.13, −0.02]).

**Conclusions:**

Prenatal anxiety is a distinct and clinically meaningful predictor of early mother–infant bonding difficulties. Its effects operate through persistent anxiety and heightened psychosocial stress, while perceived social support serves as an important protective factor. Interventions targeting prenatal anxiety, psychosocial stressors, and social support may help promote healthier mother–infant bonding in resource-constrained settings.

## Impact statement

This study identifies prenatal anxiety as a distinct and developmentally significant risk factor for early mother–infant bonding difficulties in a low-resource setting. By demonstrating that these difficulties emerge during pregnancy through modifiable psychosocial pathways and are attenuated by perceived social support, the findings shift the focus from postnatal treatment to antenatal prevention. Importantly, the results provide actionable evidence for integrating anxiety screening and psychosocial support within routine antenatal care, aligned with the World Health Organization (WHO) Health System Building Blocks framework, including service delivery, workforce task-sharing and strengthened health information systems. This work advances scalable, system-oriented approaches to maternal mental health and early relational health in low- and middle-income countries.

## Introduction

Mother–infant bonding is a core developmental process that underpins early caregiving, maternal sensitivity and infant socioemotional development. Disruptions in bonding are associated with persistent maternal distress, impaired parent–child interaction and adverse child developmental outcomes (Stuijfzand et al., 2020; Nakić Radoš et al., 2024). Despite its importance, bonding is most often conceptualized as a postpartum phenomenon, with limited attention to the prenatal psychological and social processes that shape relational readiness before birth (McNamara et al., 2019).

Perinatal mental health research has traditionally prioritized depression as the principal risk factor for impaired bonding, frequently positioning anxiety as secondary or comorbid. This framing is increasingly inadequate. Anxiety is highly prevalent during pregnancy, with estimates ranging from approximately 10%–20% in high-income countries and substantially higher rates reported in low- and middle-income settings, including South Asia (Fisher et al., 2012; Dennis et al., 2017). In Pakistan, studies indicate that up to one-third of pregnant women experience clinically significant anxiety symptoms (Gul et al., 2019; Nielsen-Scott et al., 2022). Characterized by anticipatory threat, excessive worry and heightened vigilance, prenatal anxiety directly implicates processes central to the formation of maternal expectations, emotional availability and early representations of the infant (Fairbrother et al., 2016).

Globally, the burden of prenatal anxiety is disproportionately concentrated in low- and middle-income countries, particularly in South Asia (Roddy Mitchell et al., 2023). In Pakistan, perinatal anxiety is highly prevalent and often co-occurs with socioeconomic insecurity, restricted reproductive autonomy, gendered role expectations and elevated exposure to interpersonal violence (Fisher et al., 2012; Rahman et al., 2016; Atif et al., 2021). Unlike many high-income settings, anxiety during pregnancy in these contexts frequently remains unrecognized and untreated, increasing the likelihood that its effects extend beyond maternal symptoms to influence early relational processes (Atif et al., 2020). These structural and sociocultural conditions position Pakistan as a critical context for examining prenatal anxiety not merely as a concurrent condition, but as an early psychological vulnerability with downstream consequences for mother–infant bonding (Ahmad et al., 2022; Siddiqui, 2025).

The effects of prenatal anxiety are unlikely to operate in isolation. Pregnancy unfolds within complex psychosocial environments that can either support or undermine maternal psychological adaptation (McNamara et al., 2019). Elevated anxiety has been associated with more negative pregnancy experiences, heightened perceived stress and increased exposure to interpersonal adversity, including domestic violence (Goldstein et al., 2021; Nazir et al., 2022). These psychosocial stressors represent plausible mechanisms through which prenatal psychological vulnerability may become embedded in early relational outcomes (Glover et al., 2018). By disrupting emotional safety, depleting psychological resources and shaping maladaptive expectations of motherhood, such stressors may interfere with the psychological transition required for the development of a secure maternal–infant bond (Ali et al., 2023).

Although psychosocial adversity has been linked to postpartum maternal outcomes, it is typically treated as a background risk or statistical covariate rather than as an explanatory pathway (Miller et al., 2017). As a result, existing research provides limited insight into how prenatal anxiety translates into bonding difficulties (Göbel et al., 2018). In parallel, continuity of anxiety from pregnancy into the postpartum period remains insufficiently integrated into bonding models. Longitudinal studies indicate that anxiety often persists across the perinatal period, reflecting stable vulnerability rather than transient pregnancy-related distress (Fairbrother et al., 2016). Persistent anxiety may compromise maternal emotional availability and early relational engagement, suggesting that bonding difficulties may be shaped both by ongoing symptoms and by processes initiated during pregnancy (Steen et al., 2013).

Within this broader context of vulnerability, perceived social support may function as a key protective factor, but its role is more complex than is often acknowledged. Social support is commonly examined as a direct correlate of improved outcomes, rather than as a factor that modifies risk pathway (Uchino, 2006). This distinction is particularly critical in collectivist societies such as Pakistan, where structural support from extended family systems may coexist with emotional strain, relational conflict or limited psychological safety (Hussain and Usman, 2025; Sharif and Sabir, 2025). Whether perceived social support can buffer the effects of prenatal anxiety and psychosocial stressors on early mother–infant bonding remains insufficiently examined, particularly within longitudinal designs.

Despite the high burden of perinatal psychological distress in Pakistan and similar settings, longitudinal studies integrating prenatal anxiety, psychosocial mechanisms and early bonding within a single explanatory framework are scarce. Existing research is predominantly cross-sectional, focused on depression or does not examine mediating and moderating processes that could inform prevention (Maselko et al., 2015). This gap limits the development of developmentally timed and contextually grounded interventions.

The present prospective longitudinal study addresses these limitations by testing an integrated model of prenatal psychological vulnerability and early relational outcomes. Specifically, it examines prenatal anxiety as an early driver of postpartum mother–infant bonding difficulties, independent of depressive symptoms; evaluates continuity of anxiety and late-pregnancy psychosocial stressors including pregnancy experiences, perceived stress and domestic violence as mediating mechanisms and tests perceived social support as both a direct protective factor and a moderator of these pathways. By clarifying when and how prenatal anxiety shapes early bonding in a South Asian context, this study seeks to inform preventive, socially grounded maternal mental health strategies in resource-constrained settings.

## Methods

### Study design

The quantitative component of the study employed a prospective, hospital-based longitudinal design to examine temporal associations between perinatal anxiety and maternal–infant bonding across pregnancy and the early postpartum period. Data were collected at three time points: early pregnancy (T1; 15–20 weeks gestation), late-pregnancy (T1.5; 30–36 weeks gestation) and postpartum follow-up (T2; approximately 8 weeks after delivery). This design enabled the assessment of temporal precedence, examination of psychosocial mediators measured in late-pregnancy and testing of moderation effects influencing postpartum bonding outcomes.

### Study setting and participants

Participants were recruited from the obstetrics and gynecology outpatient departments of Benazir Bhutto Hospital, Rawalpindi and Federal General Hospital, Islamabad, two large public-sector tertiary hospitals serving socioeconomically diverse populations. These settings facilitated access to pregnant women from varied educational, financial and family backgrounds, enhancing the representativeness of the sample.

Women attending routine antenatal visits were approached consecutively and screened for eligibility. Inclusion criteria were: age ≥ 18 years, pregnancy at or before 22 weeks gestation at baseline, literacy in Urdu and residence within the hospital catchment area to facilitate follow-up. Exclusion criteria included severe or life-threatening medical conditions, active suicidal ideation, a history of psychotic disorders or current use of psychotropic medication at recruitment.

### Sample size estimation

Sample size estimation was conducted using G*Power 3.1 for multiple regression and Structural Equation Modeling (SEM) analyses. Assuming a small-to-moderate effect size (*f^2^* = .05), *α* = .05, power = .95 and up to ten predictors, a minimum sample of approximately 450 participants was required. Anticipating attrition rates of 30%–35%, consistent with longitudinal perinatal research in similar contexts, a target baseline sample of approximately 700 participants was established to ensure adequate power at postpartum follow-up.

### Recruitment and data collection procedures

**Figure 1.**
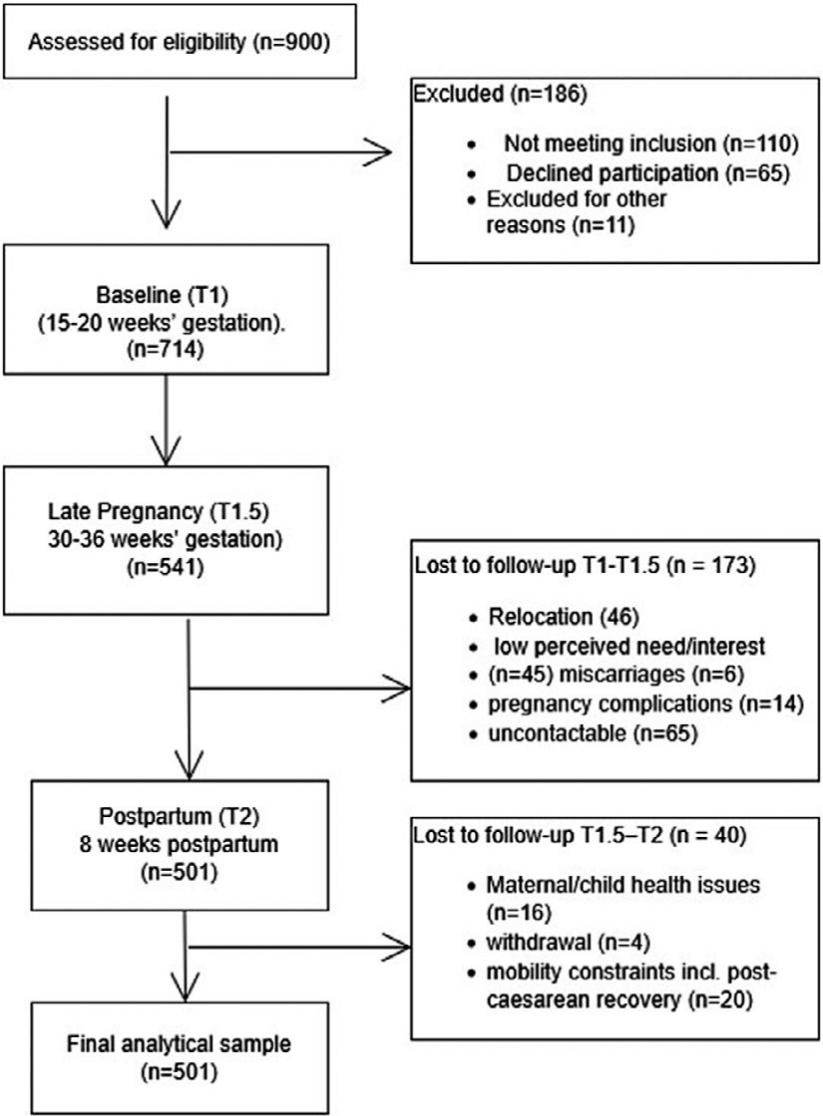
Participant flow diagram. Figure 1. long description.

Participant recruitment and follow-up are summarized in the participant flow diagram (Figure 1), presented in accordance with STROBE guidelines (von Elm et al., 2007). Of the 900 women assessed for eligibility at baseline, 714 participants completed the early pregnancy assessment (T1). Late-pregnancy follow-up (T1.5) was completed by 541 participants, and 501 women completed the postpartum assessment (T2), forming the final analytical sample.

Written informed consent was obtained at baseline following a detailed explanation of study procedures. Baseline data were collected in private consultation rooms using paper-based questionnaires administered by trained research staff and required approximately 30–40 min to complete.

Late-pregnancy assessments were conducted either during scheduled antenatal visits or via telephone when in-person follow-up was not feasible. Postpartum assessments were conducted approximately eight weeks after delivery (±2 weeks), primarily via telephone interviews. Multiple contact attempts were made at different times and days to maximize retention. Participants completing the postpartum assessment received a small non-monetary incentive.

Participants were classified as lost to follow-up if they could not be reached after repeated attempts, withdrew consent or were unavailable due to medical or logistical reasons. Attrition analyses indicated no statistically significant differences between retained and non-retained participants on baseline sociodemographic characteristics or psychological variables, suggesting minimal attrition bias.

### Measurement timeline

Anxiety and depressive symptoms were assessed at T1 and T2, allowing examination of symptom continuity and change. Psychosocial mediators and moderators, including perceived stress, pregnancy-related experiences, domestic violence exposure and perceived social support, were assessed at T1.5 to establish temporal sequencing. Maternal–infant bonding was assessed only at T2, reflecting its conceptualization as a postpartum outcome.

Infant characteristics (birth weight, birth length, infant sex) were recorded at postpartum for descriptive and sensitivity analyses but were not included as primary predictors.

### Measures

All measures were administered in Urdu, using versions that had been previously translated, culturally adapted and validated for use in Pakistani populations. Minor linguistic adjustments were made following pilot testing with 15–20 participants not included in the final sample.

### Perinatal anxiety

Anxiety symptoms were assessed using the Generalized Anxiety Disorder Scale–7 (GAD-7) (Spitzer et al., 2006). The scale comprises seven items rated on a four-point Likert scale (0–3), yielding total scores from 0 to 21, with higher scores indicating greater anxiety severity. A cutoff score of ≥10 was used to indicate clinically significant anxiety.

The Urdu version of the GAD-7, validated for Pakistani populations (Ahmad et al., 2017), demonstrated strong internal consistency and construct validity. The scale was administered at T1 and T2 to assess symptom stability and change over time.

### Depressive symptoms

Depressive symptoms were measured using the Patient Health Questionnaire–9 (PHQ-9) (Kroenke et al., 2001). The PHQ-9 includes nine items rated from 0 (“not at all”) to 3 (“nearly every day”), with total scores ranging from 0 to 27. A cutoff score of ≥10 was used to indicate probable depression. The Urdu version of the PHQ-9, validated among Pakistani pregnant women (Gallis et al., 2018), was administered at T1 and T2. Depressive symptoms were included as a correlated construct with anxiety and as a covariate in longitudinal models.

### Maternal–infant bonding

Maternal–infant bonding was assessed using the Postpartum Bonding Questionnaire (PBQ) (Brockington et al., 2001). The PBQ consists of 25 items rated on a five-point Likert scale, producing total scores from 0 to 125, with higher scores indicating greater bonding difficulties.

The Urdu version of the PBQ, culturally adapted and validated for Pakistani mothers (Naseem et al., 2025), demonstrated strong internal consistency. The total PBQ score was used as a continuous outcome variable at T2.

### Perceived social support

Perceived social support was measured using the Multidimensional Scale of Perceived Social Support (MSPSS) (Zimet et al., 1988). The scale assesses perceived support from family, friends and significant others. The Urdu-validated version (Sharif et al., 2021) was administered at T1.5 and T2. Higher scores indicate greater perceived support. Based on qualitative findings, social support was modeled as both a direct predictor and a moderator.

### Perceived stress

Perceived stress was assessed using the Perceived Stress Scale–10 (PSS-10) (Cohen et al., 1983). Total scores range from 0 to 40, with higher scores indicating greater perceived stress. The Urdu version, validated in Pakistani maternal populations (Mushtaq and Ahmad, 2020), was administered at T1.5.

### Pregnancy-related experiences

Pregnancy experiences were measured using the Pregnancy Experience Scale–Brief (PES-Brief) (Dipietro et al., 2008), assessing both positive (uplifts) and negative (hassles) aspects of pregnancy. The Urdu-validated version (Zaidi et al., 2022) was administered at T1.5. Composite scores were computed and modeled as psychosocial mediators.

### Exposure to domestic violence

Exposure to domestic violence was assessed using items adapted from the Pakistan Demographic and Health Survey Domestic Violence Module (Hassan et al., 2020). Items assessed emotional and physical violence, with higher summed scores indicating greater exposure. Domestic violence was modeled as a psychosocial risk factor in mediation analyses.

### Statistical analysis

Analyses were conducted using R (R Core Team, 2024). Data screening included assessment of missingness, outliers and distributional assumptions. Analyses were based on complete-case data (final sample *n* = 501).

Descriptive statistics were computed for all variables. Attrition analyses compared retained and non-retained participants using t-tests and chi-square tests. Longitudinal regression models examined associations between early pregnancy anxiety and postpartum bonding, adjusting for depressive symptoms and covariates. Mediation analyses employed bootstrapped confidence intervals (5,000 resamples), and moderation effects were tested using interaction terms. An integrated path model was estimated using SEM, with model fit evaluated using χ^2^, Comparative Fit Index (CFI), Tucker–Lewis Index (TLI), Root Mean Square Error of Approximation (RMSEA) and Standardized Root Mean Square Residual (SRMR).

## Results

### Participant flow, attrition and sample characteristics

Participant flow is summarized in Figure 1. Of 900 women assessed, 714 completed the baseline (T1; 15–20 weeks gestation). At postpartum (T2; 8 weeks), 501 provided outcome data (70.2% retention). Comparisons between participants retained at T2 and those lost to follow-up revealed no significant differences in baseline sociodemographic or psychological characteristics (all *p* > .05), indicating minimal attrition bias (Table 1).

**Table 1.** Sample characteristics, psychosocial variables and attrition analysis across study waves Table 1. long description.

Characteristic	Full baseline sample (*N* = 714)	Retained at T2 (*n* = 501)	Lost to follow-up (*n* = 213)	Test statistic (*p*)
**Sociodemographic (T1)**				
Maternal age, years, M (SD)	28.4 (4.7)	28.4 (4.7)	27.9 (5.1)	*t*(712) = 1.15, *p* = .251
Monthly household income (PKR), M (SD)	45,200 (15,500)	45,200 (15,500)	43,800 (16,200)	*t*(712) = 1.12, *p* = .264
Maternal education, *n* (%)				χ^2^(4) = 2.45, *p* = .294
No formal schooling	74 (10.4)	52 (10.4)	22 (10.3)	
Primary/Middle	120 (16.8)	94 (18.8)	26 (12.2)	
Secondary (Matric)	331 (46.3)	219 (43.7)	112 (52.6)	
Intermediate	88 (12.4)	65 (13.0)	23 (10.8)	
Graduate or above	101 (14.1)	71 (14.2)	30 (14.1)	
**Psychological (T1)**				
Anxiety (GAD-7), M (SD)	8.5 (4.9)	8.5 (4.9)	8.9 (5.2)	*t*(712) = 1.65, *p* = .100
Clinical anxiety (GAD-7 ≥ 10), *n* (%)	206 (28.8)	151 (30.1)	55 (25.8)	χ^2^(1) = 1.37, *p* = .242
Depression (PHQ-9), M (SD)	7.2 (4.3)	7.2 (4.3)	7.6 (4.6)	*t*(712) = 1.42, *p* = .156
**Psychosocial (T1.5)**				
Pregnancy experiences (PES-Brief), M (SD)		22.3 (7.3)		
Domestic violence (DHS-10), M (SD)		1.8 (2.5)		
Perceived stress (PSS-10), M (SD)		18.4 (6.1)		
Social support (MSPSS), M (SD)		3.42 (0.70)		
**Postpartum outcomes (T2)**				
Bonding difficulties (PBQ), M (SD)		32.1 (11.4)		
Anxiety (GAD-7), M (SD)		7.8 (4.7)		
Clinical anxiety (GAD-7 ≥ 10), *n* (%)		112 (22.4)		

*Notes:* T1 = 15–20 weeks gestation; T1.5 = 30–36 weeks gestation; T2 = 8 weeks postpartum. Percentages are column-based. PKR: Pakistani Rupees; GAD-7: Generalized Anxiety Disorder-7 (clinical cut-off ≥10); PHQ-9: Patient Health Questionnaire-9; PES-Brief: Pregnancy Experience Scale–Brief; DHS-10: Domestic Violence Scale; PSS-10: Perceived Stress Scale; MSPSS: Multidimensional Scale of Perceived Social Support; PBQ: Postpartum Bonding Questionnaire. Independent-samples t-tests and chi-square tests compared baseline (T1) characteristics between participants retained at T2 and those lost to follow-up. Psychosocial variables assessed at T1.5 were available only for participants retained at T2. No significant attrition differences were observed (*p* > .05).

Descriptive statistics are reported in Table 1. The mean maternal age was 28.4 years (*SD* = 4.7). At baseline, mean anxiety (GAD-7: *M* = 8.5, *SD* = 4.9) and depressive symptoms (PHQ-9: M = 7.2, *SD* = 4.3) were in the mild range, with 28.8% meeting the clinical cutoff for anxiety. At late-pregnancy (T1.5), participants reported moderate levels of psychosocial stressors alongside moderate-to-high perceived social support. At T2, mean mother–infant bonding difficulty (PBQ) was 32.1 (*SD* = 11.4), and 22.4% of women had clinically significant anxiety.

### Bivariate associations

Bivariate correlations are presented in Table 2. Prenatal anxiety (T1) was positively correlated with postpartum bonding difficulties (*r* = .32, *p* < .01) and postpartum anxiety (*r* = .45, *p* < .01). Prenatal depression was also correlated with postpartum bonding (*r* = .28, *p* < .01). All late-pregnancy psychosocial stressors were positively correlated with both prenatal anxiety and bonding difficulties, while perceived social support showed negative correlations.

**Table 2.** Bivariate correlations among primary study variables Table 2. long description.

Variable	1	2	3	4	5	6	7	8
1. T1 Anxiety (GAD-7)	–							
2. T1 Depression (PHQ-9)	.65**	–						
3. T1.5 Pregnancy Experiences	.28**	.25**	–					
4. T1.5 Domestic Violence	.25**	.22**	.18**	–				
5. T1.5 Perceived Stress	.35**	.31**	.25**	.22**	–			
6. T1.5 Social Support	−.31**	−.29**	−.22**	−.19**	−.28**	–		
7. T2 Bonding (PBQ)	.32**	.28**	.24**	.21**	.29**	−.26**	–	
8. T2 Anxiety (GAD-7)	.45**	.40**	.20**	.18**	.25**	−.20**	.35**	–

*Notes:* Pearson correlation coefficients are reported. *N* = 501. T1 = 15–20 weeks gestation; T1.5 = 30–36 weeks gestation; T2 = 8 weeks postpartum. GAD-7: Generalized Anxiety Disorder 7; PHQ-9: Patient Health Questionnaire–9; PBQ: Postpartum Bonding Questionnaire. **p < .01 (two-tailed).

### Longitudinal, mediation and moderation pathways

A longitudinal path model (Table 3, Panel A) demonstrated excellent fit. Prenatal anxiety had a significant direct effect on postpartum bonding difficulties (*β* = .18, *p* = .001) and a strong indirect effect mediated through postpartum anxiety. Prenatal depression was not a significant predictor when modeled concurrently.

**Table 3.** Direct, indirect and moderated pathways linking prenatal anxiety to postpartum bonding Table 3. long description.

Panel A. longitudinal path model (standardized estimates)			
Path	β	SE	*p*
T1 Anxiety → T2 Bonding	0.18	0.05	0.001
T1 Anxiety → T2 Anxiety	0.45	0.04	< 0.001
T2 Anxiety → T2 Bonding	0.28	0.05	< 0.001
T1 Depression → T2 Bonding	0.05	0.05	0.312
T1 Anxiety ↔ T1 Depression (r)	0.65	0.03	< 0.001

Model fit indices	
Index	Value
χ^2^(df), *p*	3.15 (2), 0.207
CFI / TLI	0.99 / 0.98
RMSEA [90% CI]	0.043 [0.000, 0.124]
SRMR	0.019

Panel B. parallel mediation analysis (unstandardized effects)			
Effect	B	Boot SE	95% CI
Total effect (c)	0.86	0.10	[0.67, 1.05]
Direct effect (c′)	0.52	0.10	[0.33, 0.71]
Total indirect effect (ab)	0.34	0.06	[0.23, 0.46]
Via Pregnancy Experiences	0.11	0.04	[0.04, 0.19]
Via Domestic Violence	0.08	0.03	[0.03, 0.15]
Via Perceived Stress	0.15	0.04	[0.08, 0.23]

Panel C. moderation by social support (unstandardized effects)				
Predictor	B	SE	*p*	95% CI
T1 Anxiety	0.68	0.08	< 0.001	[0.52, 0.84]
T1.5 Social Support	−0.19	0.04	< 0.001	[−0.27, −0.11]
Anxiety × Social Support	−0.07	0.02	< 0.001	[−0.11, −0.03]

*Notes: N* = 501. T1 = 15–20 weeks gestation; T1.5 = 30–36 weeks gestation; T2 = 8 weeks postpartum. Panel A reports standardized path coefficients (β); model fit indices: CFI: Comparative Fit Index; TLI = Tucker–Lewis Index; RMSEA = Root Mean Square Error of Approximation; SRMR = Standardized Root Mean Square Residual. Panels B and C report unstandardized regression coefficients (B). Mediation effects (Panel B) tested using 5,000 bootstrap resamples; CI = bias-corrected confidence interval. Panel C model R^2^ = 0.198, F (7, 493) = 17.32, *p* < 0.001; predictors were mean-centered.

**Figure 2.**
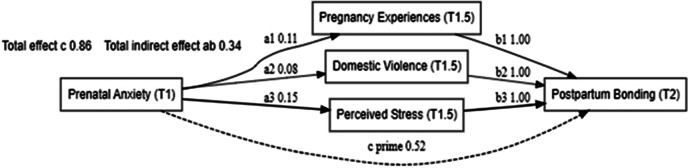
Parallel multiple mediation model of prenatal anxiety on postpartum bonding. Figure 2. long description.

Parallel mediation analysis (Table 3, Panel B and Figure 2) indicated that late-pregnancy psychosocial stressors (pregnancy experiences, domestic violence, perceived stress) partially mediated the prenatal anxiety-bonding link (total indirect effect = 0.34, 95% CI [0.23, 0.46]). The direct effect remained significant (c′ = 0.52, *p* < .001).

**Figure 3.**
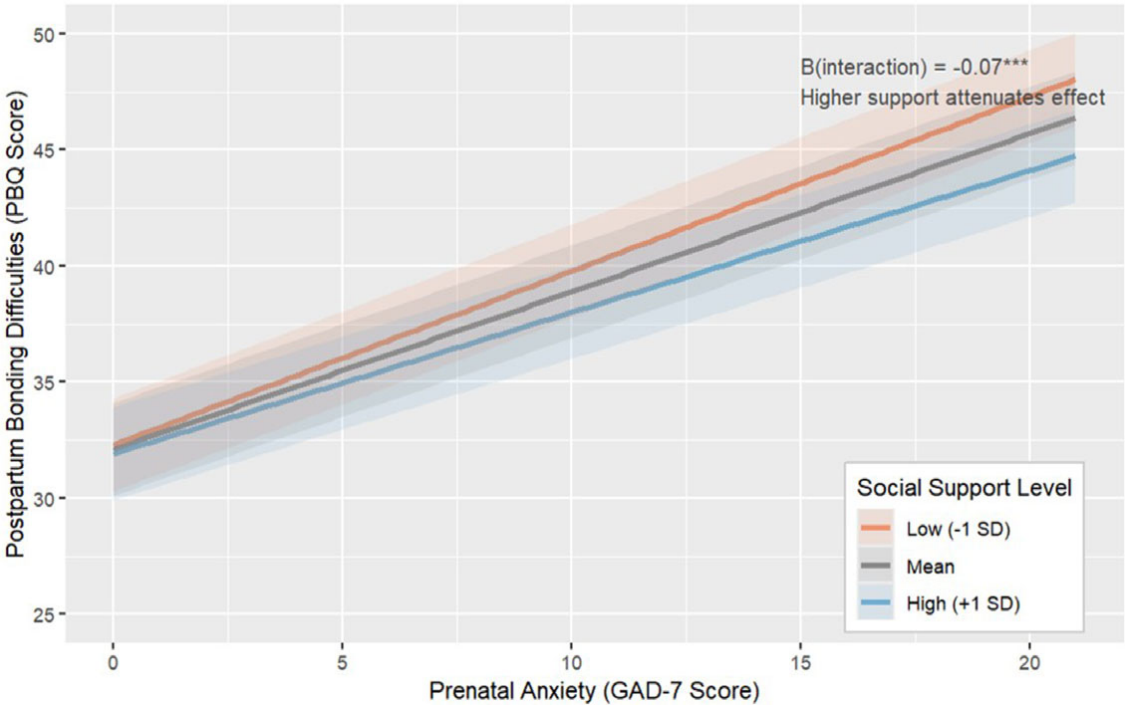
Moderation of the anxiety-bonding relationship by perceived social support. Figure 3. long description.

Moderated regression (Table 3, Panel C and Figure 3) showed that perceived social support (T1.5) significantly buffered the direct association between prenatal anxiety and bonding (interaction *B* = −0.07, *p* < .001). The association was strongest at low levels of support.

### Integrated model and conditional indirect effects

An integrated SEM confirmed these pathways (Table 4), explaining 44% of the variance in bonding difficulties. The model reaffirmed the direct effect of prenatal anxiety (*β* = .17, *p* = .001), the significant paths from psychosocial stressors and the protective direct (*β* = −.24, *p* < .001) and moderating (*β* = −.13, *p* = .001) roles of social support.

**Table 4.** Integrated structural equation model: Standardized path estimates Table 4. long description.

Path	β	SE	*p*
**Anxiety pathways**			
T1 Anxiety → T2 Bonding	.17	.05	.001
T1 Anxiety → T2 Anxiety	.45	.04	< .001
T2 Anxiety → T2 Bonding	.28	.05	< .001
**Late-pregnancy psychosocial pathways**			
T1.5 Pregnancy Stress → T2 Bonding	.21	.06	.002
T1.5 Domestic Violence → T2 Bonding	.16	.05	.004
**Social support pathways**			
T1.5 Social Support → T2 Bonding	−.24	.06	< .001
T1 Anxiety × T1.5 Social Support → T2 Bonding	−.13	.04	.001
**Model summary**			
χ^2^(df), *p*	28.42 (18)		.056
CFI / TLI	.98 / .97		
RMSEA [90% CI]	.035 [.000, .062]		
SRMR	.029		
R^2^ for T2 Bonding	.44		

*Notes:* N = 501. T1 = 15–20 weeks gestation; T1.5 = 30–36 weeks gestation; T2 = 8 weeks postpartum. All coefficients are standardized (β). Model fit indices: CFI: Comparative Fit Index; TLI: Tucker–Lewis Index; RMSEA: Root Mean Square Error of Approximation; SRMR: Standardized Root Mean Square Residual.

The moderated mediation was probed further (Table 5). The indirect effects of prenatal anxiety on bonding (via pregnancy experiences and domestic violence) were strongest at low levels of social support and attenuated at higher levels (Index of Moderated Mediation = −0.07, 95% CI [−0.13, −0.02]).

**Table 5.** Conditional indirect effects of prenatal anxiety on postpartum bonding at levels of social support Table 5. long description.

Level of social support (MSPSS)	Indirect effect via pregnancy experiences	95% CI	Indirect effect via domestic violence	95% CI
Low (−1 *SD*)	0.18	[0.09, 0.28]	0.12	[0.05, 0.20]
Mean	0.11	[0.04, 0.19]	0.08	[0.03, 0.15]
High (+1 *SD*)	0.04	[0.01, 0.08]	0.04	[0.01, 0.08]

*Notes: N* = 501. Unstandardized coefficients are reported. Conditional indirect effects were estimated using 5,000 bootstrap resamples. CI: bias-corrected confidence interval. Social support was measured at T1.5 (30–36 weeks gestation). The index of moderated mediation was −0.07, 95% CI [−0.13, −0.02].

### Sensitivity analyses

Sensitivity analyses confirmed the robustness of the primary association between prenatal anxiety (T1) and postpartum bonding (T2). The effect remained stable (standardized β ~ .16–.19, all p ≤ .003) when using alternative anxiety cutoffs (GAD-7 ≥ 8, ≥10), excluding prenatal depressive symptoms and adjusting for infant birth characteristics (weight and length) see Supplementary Table 1.

## Discussion

This prospective longitudinal study demonstrates that prenatal anxiety is a central and early driver of postpartum mother–infant bonding difficulties, operating through both direct and indirect psychosocial pathways and conditionally shaped by perceived social support. Anxiety in early pregnancy predicted poorer bonding at eight weeks postpartum, independent of prenatal depressive symptoms. This association was partially explained by continuity of anxiety into the postpartum period and by late-pregnancy psychosocial stressors, including negative pregnancy experiences, perceived stress and exposure to domestic violence. At the same time, perceived social support emerged as a robust protective factor, showing both a direct association with improved bonding and a moderating effect that buffered the impact of prenatal anxiety. Together, these pathways accounted for a substantial proportion of variance in bonding difficulties, underscoring pregnancy as a sensitive period during which psychological vulnerability and social context jointly shape early relational outcomes.

Taken together, the findings advance perinatal mental health research in three ways. First, they position prenatal anxiety – rather than depression alone – as a developmentally salient exposure for early bonding. Second, they demonstrate that late-pregnancy psychosocial stressors function as explanatory mechanisms rather than background correlates. Third, they provide evidence that social support not only improves outcomes on average but actively alters the strength of risk pathways, including indirect effects.

The finding that prenatal anxiety independently predicted postpartum bonding difficulties reinforces emerging evidence that anxiety during pregnancy has distinct consequences for maternal–infant relationships. While prior research has focused largely on postpartum depression as the primary psychological risk for bonding (Ohara et al., 2017), fewer studies have examined anxiety prospectively or modeled it alongside depression (Dubber et al., 2015). The present results align with longitudinal work linking antenatal anxiety to later difficulties in maternal sensitivity, emotional availability and bonding representations (Steen et al., 2013; Dubber et al., 2015; Naseem et al., 2025).

While the observed association between prenatal anxiety and bonding difficulties is consistent with prior longitudinal findings (Steen et al., 2013; Naseem et al., 2025), the strength and configuration of these pathways in the present study suggest important contextual differences. In high-income settings, the effects of antenatal anxiety on early relational outcomes are often examined within contexts characterized by structured perinatal care and greater access to psychosocial support (Fairbrother et al., 2016). In contrast, in Pakistan and similar low-resource settings, anxiety is embedded within conditions of socioeconomic constraint, limited access to mental health services and heightened exposure to psychosocial adversity, including interpersonal violence and chronic stress (Rahman et al., 2016; Nazir et al., 2022). These contextual conditions may amplify the impact of anticipatory distress, increasing its salience for maternal emotional availability and early bonding processes.

Moreover, the prominence of psychosocial mediators in the present study, particularly perceived stress and domestic violence, reflects patterns more characteristic of low- and middle-income contexts, where adversity is not peripheral but central to the perinatal experience (Glover et al., 2018). This contrasts with much of the existing literature, where such variables are frequently treated as covariates rather than as explanatory pathways (Miller et al., 2017). The findings therefore extend prior work by demonstrating that prenatal anxiety operates not only as an individual psychological vulnerability but as part of a broader socio-structural risk system shaping early mother–infant relationships.

Notably, prenatal depressive symptoms did not independently predict bonding once anxiety was included in the models. This does not suggest that depression is unimportant, but rather that the anticipatory threat, uncertainty and hypervigilance characteristic of anxiety may be especially disruptive to the psychological processes involved in forming early relational expectations. In settings marked by medical uncertainty, economic strain and limited institutional support, such anticipatory distress may be particularly salient, rendering anxiety a more potent predictor of bonding difficulties than low mood alone.

Consistent with previous longitudinal studies, prenatal anxiety strongly predicted postpartum anxiety, which in turn was associated with poorer bonding outcomes (Kaydırak et al., 2022; Le Bas et al., 2022). This pattern suggests that anxiety during pregnancy often reflects a persistent vulnerability rather than a transient response to pregnancy-related changes.

Postpartum anxiety partially mediated the association between prenatal anxiety and bonding difficulties, indicating that sustained maternal anxiety compromises early relational engagement. However, the persistence of a significant direct effect of prenatal anxiety after accounting for postpartum symptoms suggests that important processes are initiated during pregnancy itself. This finding supports theoretical models that conceptualize bonding as rooted in antenatal psychological adaptation, rather than emerging solely in response to postnatal interactions.

Late-pregnancy psychosocial stressors accounted for a meaningful portion of the association between prenatal anxiety and postpartum bonding, clarifying how early psychological vulnerability becomes embedded in relational outcomes. Women with higher prenatal anxiety reported more negative pregnancy experiences, greater perceived stress and higher exposure to domestic violence, each of which independently contributed to bonding difficulties.

Negative pregnancy experiences may interfere with the psychological transition to motherhood by shaping maladaptive expectations and emotional disengagement from the maternal role (Dipietro et al., 2008; Carpinelli and Savarese, 2022). Elevated perceived stress likely reflects cumulative demands that erode emotional resources, reducing the capacity for sensitive caregiving in the early postpartum period. The mediating role of domestic violence is particularly concerning but consistent with evidence linking prenatal exposure to interpersonal threat with impaired maternal functioning and relational insecurity (Kita et al., 2016; Naseem et al., 2025). In this context, violence likely operates both as a direct source of trauma and as a marker of chronic relational instability.

By modeling these factors as mediators rather than covariates, the study demonstrates that psychosocial adversity is not merely correlated with bonding difficulties but constitutes part of the pathway through which prenatal anxiety exerts its effects.

Perceived social support emerged as one of the most influential factors in the model. Higher support was associated with fewer bonding difficulties and significantly moderated the association between prenatal anxiety and bonding outcomes. The interaction indicated that prenatal anxiety was most strongly associated with bonding difficulties among women reporting low levels of support, whereas this association was substantially attenuated at higher support levels.

These findings are consistent with stress-buffering theory (Cohen and Wills, 1985) and with perinatal research demonstrating protective effects of social support on maternal mental health and caregiving behaviors (Balaji et al., 2007; Hirani, 2017). Importantly, moderated mediation analyses showed that social support weakened indirect pathways through pregnancy experiences and domestic violence, suggesting that supportive environments can disrupt the translation of psychological vulnerability into relational harm.

Overall, this study identifies prenatal anxiety as a pivotal and independent risk factor for postpartum bonding difficulties, operating through a cascade of sustained maternal symptoms and late-pregnancy psychosocial adversity. The robust moderating role of perceived social support underscores that this risk pathway is malleable. The findings move beyond establishing correlation to illuminate a modifiable psychological and social process: bonding is shaped during pregnancy, not just after birth. This underscores a critical window for prevention. Perinatal mental health care should therefore prioritize early screening for anxiety, alongside efforts to mitigate concurrent stressors and, most importantly, to cultivate the quality of the mother’s social environment, transforming it from a potential source of strain into a confirmed buffer for the developing mother–infant relationship.

### Strengths

This study is strengthened by its prospective design with temporally ordered assessments, allowing prenatal anxiety (T1), late-pregnancy psychosocial processes (T1.5) and postpartum bonding outcomes (T2) to be modeled with clear temporal precedence. The large sample and postpartum retention rate exceeding 70% compare favorably with longitudinal perinatal studies in similar settings, and attrition analyses suggested minimal bias. All measures were Urdu-validated instruments with demonstrated psychometric adequacy in Pakistani maternal populations. The convergence of findings across longitudinal regression, mediation, moderation and SEM further strengthens confidence in the robustness of the results.

### Limitations

Several limitations warrant consideration. Bonding was assessed using self-report rather than observational methods. While this may introduce reporting bias, subjective bonding experiences are conceptually central to the construct and predictive of later relational functioning. Although depressive symptoms were adjusted for, other psychological factors such as trauma history or pregnancy-specific fears were not assessed and may partially overlap with anxiety. Infant-related factors beyond basic birth characteristics were not included in primary models. Finally, recruitment from public-sector hospitals may limit generalizability to women receiving private care; however, these settings reflect the care context of the majority of pregnant women in Pakistan.

### Implications for research and practice

The findings identify pregnancy as a critical window for prevention and clarify that intervention efforts must extend beyond symptom reduction. Prenatal anxiety influenced bonding through modifiable psychosocial pathways, including perceived stress, negative pregnancy experiences and domestic violence, indicating the need for integrated antenatal approaches that address both emotional regulation and contextual stressors. The buffering effect of perceived social support further highlights the importance of strengthening protective environments. In collectivist contexts, where support is often structurally available but not consistently emotionally protective, the quality of support becomes central. Interventions that enhance supportive interactions and reduce relational strain may therefore play a key role in improving early mother–infant bonding.

### Health system implementation considerations

Translating these findings into practice requires alignment with health system priorities in low-resource settings. Using the World Health Organization (WHO) Health System Building Blocks framework (2007), several implementation considerations emerge.

### Service delivery

Routine screening for anxiety should be integrated into antenatal care using brief, validated tools such as the GAD-7 (Spitzer et al., 2006). Given that psychosocial stressors partially mediate the association with bonding difficulties, screening should be accompanied by brief psychosocial interventions that address stress, safety and pregnancy-related experiences rather than focusing solely on symptom reduction.

### Health workforce

Task-sharing approaches are essential in contexts with limited specialist capacity. Non-specialist providers, including Lady Health Workers and mid-level maternal care staff, can be trained to deliver structured, low-intensity psychological interventions, building on existing models successfully implemented in Pakistan (Rahman et al., 2016; Atif et al., 2020).

### Health information systems

Incorporating maternal mental health indicators into routine antenatal records would allow early identification of women at risk and enable longitudinal monitoring across pregnancy and the postpartum period.

### Access to interventions

The findings support the use of scalable, low-intensity psychological interventions targeting anxiety and psychosocial stressors. These approaches are more feasible than specialist-led models and align with the need for accessible care in resource-constrained settings (Rahman et al., 2016).

### Financing

Integration within existing maternal and child health programs is likely to be more cost-effective than developing parallel mental health services. Leveraging existing service platforms can facilitate scale without substantial additional resource burden.

### Leadership and governance

At the policy level, the inclusion of maternal mental health, particularly anxiety, within national antenatal care guidelines is critical. Current frameworks often prioritize depression, and the present findings suggest that this focus may overlook a key pathway to early relational difficulties.

## Conclusions

Postpartum bonding difficulties appear to be shaped across pregnancy through interacting psychological and social processes rather than emerging solely after childbirth. Prenatal anxiety initiates a cascade involving persistent anxiety and heightened psychosocial stress, increasing vulnerability to early relational difficulties. Perceived social support can meaningfully attenuate these pathways.

These findings support a shift toward developmentally timed antenatal interventions that address both psychological vulnerability and social context. Targeting prenatal anxiety and strengthening supportive environments during pregnancy may represent effective strategies for promoting healthier early mother–infant relationships in low-resource settings.

## Supporting information

10.1017/gmh.2026.10228.sm001 Liaqat and Arouj supplementary material Liaqat and Arouj supplementary material

## Data Availability

The quantitative dataset is not publicly available due to ethical and confidentiality restrictions. De-identified data may be made available upon reasonable request to the corresponding author, subject to institutional approval.

## Supplementary material

The supplementary material for this article can be found at http://doi.org/10.1017/gmh.2026.10228.

## Long descriptions

### Figure 1. Long description

At the top, a box labeled Assessed for eligibility n equals 900 leads downward to Baseline T 1, 15 to 20 weeks’ gestation, n equals 714. From the initial box, a rightward arrow points to Excluded n equals 186, with bullet points Not meeting inclusion n equals 110, Declined participation n equals 65, Excluded for other reasons n equals 11. The main path continues downward to Late Pregnancy T 1 point 5, 30 to 36 weeks’ gestation, n equals 541. From this, a rightward arrow points to Lost to follow-up T 1 to T 1 point 5 n equals 173, with bullets Relocation 46, low perceived need or interest 45, miscarriages 6, pregnancy complications 14, uncontactable 65. The main path continues to Postpartum T 2, 8 weeks postpartum, n equals 501. A rightward arrow points to Lost to follow-up T 1 point 5 to T 2 n equals 40, with bullets Maternal or child health issues 16, withdrawal 4, mobility constraints including post-caesarean recovery 20. The final box at the bottom is Final analytical sample n equals 501.

Navigate back to Figure 1..

### Table 1. Long description

The table has five columns: characteristic, full baseline sample N equals 714, retained at T2 n equals 501, lost to follow-up n equals 213, and test statistic p. Section one, Sociodemographic T1, includes maternal age (years, mean 28.4 SD 4.7 for baseline and retained, 27.9 SD 5.1 for lost, t 712 equals 1.15, p equals point 251), monthly household income in P K R (mean 45,200 SD 15,500 for baseline and retained, 43,800 SD 16,200 for lost, t 712 equals 1.12, p equals point 264), and maternal education (chi-squared 4 equals 2.45, p equals point 294) with subcategories: no formal schooling (74, 10.4 percent baseline; 52, 10.4 percent retained; 22, 10.3 percent lost), primary or middle (120, 16.8 percent baseline; 94, 18.8 percent retained; 26, 12.2 percent lost), secondary or matric (331, 46.3 percent baseline; 219, 43.7 percent retained; 112, 52.6 percent lost), intermediate (88, 12.4 percent baseline; 65, 13.0 percent retained; 23, 10.8 percent lost), graduate or above (101, 14.1 percent baseline; 71, 14.2 percent retained; 30, 14.1 percent lost). Section two, Psychological T1, includes anxiety G A D dash 7 (mean 8.5 SD 4.9 for baseline and retained, 8.9 SD 5.2 for lost, t 712 equals 1.65, p equals point 100), clinical anxiety G A D dash 7 greater than or equal to 10 (206, 28.8 percent baseline; 151, 30.1 percent retained; 55, 25.8 percent lost, chi-squared 1 equals 1.37, p equals point 242), and depression P H Q dash 9 (mean 7.2 SD 4.3 for baseline and retained, 7.6 SD 4.6 for lost, t 712 equals 1.42, p equals point 156). Section three, Psychosocial T1.5, only for retained at T2, includes pregnancy experiences P E S dash Brief (mean 22.3 SD 7.3), domestic violence D H S dash 10 (mean 1.8 SD 2.5), perceived stress P S S dash 10 (mean 18.4 SD 6.1), and social support M S P S S (mean 3.42 SD 0.70). Section four, Postpartum outcomes T2, only for retained at T2, includes bonding difficulties P B Q (mean 32.1 SD 11.4), anxiety G A D dash 7 (mean 7.8 SD 4.7), and clinical anxiety G A D dash 7 greater than or equal to 10 (112, 22.4 percent). Test statistics show no significant differences between retained and lost groups for all baseline variables, with all p values greater than point 05. Abbreviations: P K R is Pakistani Rupees, G A D dash 7 is Generalized Anxiety Disorder dash 7, P H Q dash 9 is Patient Health Questionnaire dash 9, P E S dash Brief is Pregnancy Experience Scale dash Brief, D H S dash 10 is Domestic Violence Scale, P S S dash 10 is Perceived Stress Scale, M S P S S is Multidimensional Scale of Perceived Social Support, P B Q is Postpartum Bonding Questionnaire. Timepoints: T1 is 15 to 20 weeks gestation, T1.5 is 30 to 36 weeks gestation, T2 is 8 weeks postpartum.

Navigate back to Table 1..

### Table 2. Long description

The table contains eight variables listed vertically and horizontally: 1 T1 Anxiety G A D dash 7, 2 T1 Depression P H Q dash 9, 3 T1 point 5 Pregnancy Experiences, 4 T1 point 5 Domestic Violence, 5 T1 point 5 Perceived Stress, 6 T1 point 5 Social Support, 7 T2 Bonding P B Q, 8 T2 Anxiety G A D dash 7. The diagonal from top left to bottom right contains dashes. Off-diagonal cells report Pearson correlation coefficients, all significant at p less than point zero one, two-tailed. Notable positive correlations include T1 Anxiety with T1 Depression point six five, T1 Anxiety with T2 Anxiety point four five, T1 Anxiety with T2 Bonding point three two, and T1 Anxiety with T1 point 5 Perceived Stress point three five. T1 point 5 Social Support shows negative correlations with all other variables, strongest with T1 Anxiety negative point three one, T1 Depression negative point two nine, and T1 point 5 Perceived Stress negative point two eight. T2 Bonding is positively correlated with all variables except T1 point 5 Social Support negative point two six. All coefficients marked with double asterisks indicate significance. Sample size is N equals five hundred one. T1 is fifteen to twenty weeks gestation, T1 point 5 is thirty to thirty six weeks gestation, T2 is eight weeks postpartum. G A D dash 7 is Generalized Anxiety Disorder 7, P H Q dash 9 is Patient Health Questionnaire dash 9, P B Q is Postpartum Bonding Questionnaire.

Navigate back to Table 2..

### Figure 2. Long description

At the far left is a box labeled Prenatal Anxiety T1. Three arrows extend rightward from this box to three parallel boxes: Pregnancy Experiences T1.5 at the top, Domestic Violence T1.5 in the middle, and Perceived Stress T1.5 at the bottom. The arrows are labeled a1 0.11 to Pregnancy Experiences, a2 0.08 to Domestic Violence, and a3 0.15 to Perceived Stress. Each of these three mediator boxes has a rightward arrow pointing to a single box at the far right labeled Postpartum Bonding T2. The arrows are labeled b1 1.00 from Pregnancy Experiences, b2 1.00 from Domestic Violence, and b3 1.00 from Perceived Stress. A dashed arrow labeled c prime 0.52 connects Prenatal Anxiety T1 directly to Postpartum Bonding T2. Above the diagram, text reads Total effect c 0.86 and Total indirect effect ab 0.34.

Navigate back to Figure 2..

### Figure 3. Long description

X axis is Prenatal Anxiety (G A D dash 7 Score) from 0 to 20. Y axis is Postpartum Bonding Difficulties (P B Q Score) from 25 to 50. Three lines represent Social Support Level: top line is Low (minus 1 S D, orange), middle is Mean (gray), bottom is High (plus 1 S D, blue). All lines show a positive linear trend, but the slope is steepest for low support and shallowest for high support. Shaded bands around each line indicate confidence intervals. In the upper right, text reads B interaction equals minus 0.07, three asterisks, Higher support attenuates effect. The legend in the lower right identifies line colors for each support level.

Navigate back to Figure 3..

### Table 4. Long description

The table is organized by pathway categories. Under Anxiety pathways: T1 Anxiety to T2 Bonding beta point one seven, standard error point zero five, p equals point zero zero one; T1 Anxiety to T2 Anxiety beta point four five, standard error point zero four, p less than point zero zero one; T2 Anxiety to T2 Bonding beta point two eight, standard error point zero five, p less than point zero zero one. Under Late-pregnancy psychosocial pathways: T1 point five Pregnancy Stress to T2 Bonding beta point two one, standard error point zero six, p equals point zero zero two; T1 point five Domestic Violence to T2 Bonding beta point one six, standard error point zero five, p equals point zero zero four. Under Social support pathways: T1 point five Social Support to T2 Bonding beta negative point two four, standard error point zero six, p less than point zero zero one; T1 Anxiety times T1 point five Social Support to T2 Bonding beta negative point one three, standard error point zero four, p equals point zero zero one. Model summary indices: chi-squared twenty-eight point four two with eighteen degrees of freedom, p equals point zero five six; C F I point nine eight, T L I point nine seven; R M S E A point zero three five with ninety percent confidence interval zero to point zero six two; S R M R point zero two nine; R squared for T2 Bonding point four four. Notes: N equals five hundred one. T1 equals fifteen to twenty weeks gestation, T1 point five equals thirty to thirty-six weeks gestation, T2 equals eight weeks postpartum. All coefficients are standardized. Model fit indices: C F I is Comparative Fit Index, T L I is Tucker-Lewis Index, R M S E A is Root Mean Square Error of Approximation, S R M R is Standardized Root Mean Square Residual.

Navigate back to Table 4..

### Table 5. Long description

The table has three rows for levels of social support: Low (minus 1 S D), Mean, and High (plus 1 S D). For Low support, the indirect effect via pregnancy experiences is 0.18 with confidence interval 0.09 to 0.28, and via domestic violence is 0.12 with confidence interval 0.05 to 0.20. For Mean support, the indirect effect via pregnancy experiences is 0.11 with confidence interval 0.04 to 0.19, and via domestic violence is 0.08 with confidence interval 0.03 to 0.15. For High support, the indirect effect via pregnancy experiences is 0.04 with confidence interval 0.01 to 0.08, and via domestic violence is 0.04 with confidence interval 0.01 to 0.08. All coefficients are unstandardized. The index of moderated mediation is minus 0.07 with confidence interval minus 0.13 to minus 0.02. Social support was measured at T 1 point 5, corresponding to 30 to 36 weeks gestation. N equals 501. Conditional indirect effects were estimated using 5,000 bootstrap resamples.

Navigate back to Table 5..

### Table 3. Long description

Panel A: Longitudinal path model (standardized estimates).

* T 1 Anxiety yields T 2 Bonding: beta 0.18, S E 0.05, p 0.001.

* T 1 Anxiety yields T 2 Anxiety: beta 0.45, S E 0.04, p less than 0.001.

* T 2 Anxiety yields T 2 Bonding: beta 0.28, S E 0.05, p less than 0.001.

* T 1 Depression yields T 2 Bonding: beta 0.05, S E 0.05, p 0.312.

* T 1 Anxiety correlates with T 1 Depression: r 0.65, S E 0.03, p less than 0.001.

Model fit indices:

* Chi-squared (df) 3.15 (2), p 0.207.

* C F I / T L I: 0.99 / 0.98.

* R M S E A [90% C I]: 0.043 [0.000, 0.124].

* S R M R: 0.019.

Panel B: Parallel mediation analysis (unstandardized effects).

* Total effect (c): B 0.86, Boot S E 0.10, 95% C I [0.67, 1.05].

* Direct effect (c prime): B 0.52, Boot S E 0.10, 95% C I [0.33, 0.71].

* Total indirect effect (ab): B 0.34, Boot S E 0.06, 95% C I [0.23, 0.46].

* Via Pregnancy Experiences: B 0.11, Boot S E 0.04, 95% C I [0.04, 0.19].

* Via Domestic Violence: B 0.08, Boot S E 0.03, 95% C I [0.03, 0.15].

* Via Perceived Stress: B 0.15, Boot S E 0.04, 95% C I [0.08, 0.23].

Panel C: Moderation by social support (unstandardized effects).

* T 1 Anxiety: B 0.68, S E 0.08, p less than 0.001, 95% C I [0.52, 0.84].

* T 1.5 Social Support: B negative 0.19, S E 0.04, p less than 0.001, 95% C I [negative 0.27, negative 0.11].

* Anxiety multiplied by Social Support: B negative 0.07, S E 0.02, p less than 0.001, 95% C I [negative 0.11, negative 0.03].

Navigate back to Table 3..
