# Supplementary material for: Pathways from prenatal anxiety to postpartum bonding difficulties in Pakistan: A prospective longitudinal study of psychosocial mediators and the buffering role of social support
Source: Glob Ment Health (Camb). 2026 May 18;13:e117. doi: 10.1017/gmh.2026.10228 (PMC13244228; doi:10.1017/gmh.2026.10228)
Supplement: Liaqat and Arouj supplementary material [file S2054425126102283sup001.docx]

**Supplementary table 1**
