# Supplementary material for: Pathways from prenatal anxiety to postpartum bonding difficulties in Pakistan: A prospective longitudinal study of psychosocial mediators and the buffering role of social support
Source: Glob Ment Health (Camb). 2026 May 18;13:e117. doi: 10.1017/gmh.2026.10228 (PMC13244228; doi:10.1017/gmh.2026.10228)
Supplement: Peer review history

# Reviewer report: Review: Pathways from prenatal anxiety to postpartum bonding difficulties in Pakistan: A prospective longitudinal study of psychosocial mediators and the buffering role of social support — R0/PR2

**DOI:** 10.1017/gmh.2026.10228.pr2

This is an interesting and original paper. It shows that prenatal anxiety independently predicted postnatal bonding difficulties in Pakistani women whereas prenatal depression did not. The authors draw appropriate implications for prenatal care.

I am not in a position to evaluate the statistics.

# Reviewer report: Review: Pathways from prenatal anxiety to postpartum bonding difficulties in Pakistan: A prospective longitudinal study of psychosocial mediators and the buffering role of social support — R0/PR3

**DOI:** 10.1017/gmh.2026.10228.pr3

This is an excellent validation of the existing findings from Pakistan; please ensure in discussion that relevant evidence is compared and contrasted from other and same cultural background. Please improve the implementation section of this paper where you would like to discuss bottlenecks according to WHO Building Blocks.

# Editor report: Recommendation: Pathways from prenatal anxiety to postpartum bonding difficulties in Pakistan: A prospective longitudinal study of psychosocial mediators and the buffering role of social support — R0/PR4

**DOI:** 10.1017/gmh.2026.10228.pr4

**Affiliations:** Fudan University, China

Please address the second reviewer’s comment: ensure in discussion that relevant evidence is compared and contrasted from other and same cultural background; improve the implementation section of this paper where you would like to discuss bottlenecks according to WHO Building Blocks.

# Editor report: Decision: Pathways from prenatal anxiety to postpartum bonding difficulties in Pakistan: A prospective longitudinal study of psychosocial mediators and the buffering role of social support — R0/PR5

**DOI:** 10.1017/gmh.2026.10228.pr5

**Affiliations:** London School of Hygiene & Tropical Medicine, United Kingdom of Great Britain and Northern Ireland

No accompanying comment.

# Editor report: Recommendation: Pathways from prenatal anxiety to postpartum bonding difficulties in Pakistan: A prospective longitudinal study of psychosocial mediators and the buffering role of social support — R1/PR7

**DOI:** 10.1017/gmh.2026.10228.pr7

**Affiliations:** Fudan University, China

No accompanying comment.

# Editor report: Decision: Pathways from prenatal anxiety to postpartum bonding difficulties in Pakistan: A prospective longitudinal study of psychosocial mediators and the buffering role of social support — R1/PR8

**DOI:** 10.1017/gmh.2026.10228.pr8

**Affiliations:** London School of Hygiene & Tropical Medicine, United Kingdom of Great Britain and Northern Ireland

No accompanying comment.
